# Sleep and circadian disorders as risk factors for autoimmune disease: A population-based study

**DOI:** 10.1016/j.nbscr.2025.100129

**Published:** 2025-05-20

**Authors:** Amber R. Li, Bhaavyaa Shah, Michael L. Thomas, Michael J. McCarthy, Alejandro D. Meruelo

**Affiliations:** a University of California, 9500 Gilman Dr, La Jolla, San Diego, CA, 92093, USA; b Colorado State University, 1876 Campus Delivery, Fort Collins, CO, 80523-1876, USA; c University of California, VA San Diego Healthcare System, 3350 La Jolla Village Dr San Diego, CA, 92161, USA

**Keywords:** Chronotype, Delayed sleep phase disorder, Obstructive sleep apnea, Primary insomnia, Hypersomnia, Autoimmune disease

## Abstract

**Background:**

Sleep and circadian disruption have been increasingly linked to immune dysregulation, yet population-level associations with autoimmune disease remain underexplored. We examined whether delayed sleep phase disorder (DSPD), obstructive sleep apnea (OSA), primary insomnia, and hypersomnia were associated with autoimmune conditions in a large, diverse U.S. cohort.

**Methods:**

Data were drawn from the All of Us Research Program Registered Tier Dataset v8. Participants were categorized into sleep disorder groups based on clinical diagnoses, with regular sleepers serving as controls. Autoimmune disease was defined using SNOMED-coded records. DSPD and primary insomnia were analyzed using rare disease logistic regression; OSA and hypersomnia were analyzed using 1:5 propensity score matching. Adjusted logistic regression models included age, sex at birth, race, ethnicity, income, BMI, and chronic inflammatory diagnosis. E-values assessed robustness to unmeasured confounding.

**Results:**

All four sleep disorder groups showed significantly higher odds of autoimmune diagnosis relative to regular sleepers (p < 2.2 × 10^−16^). Adjusted odds ratios were: DSPD (OR = 0.26; 95 % CI: 0.15–0.45), OSA (OR = 0.46; 95 % CI: 0.41–0.52), primary insomnia (OR = 0.46; 95 % CI: 0.41–0.52), and hypersomnia (OR = 0.48; 95 % CI: 0.46–0.50). Older age, female sex, and chronic inflammation were associated with higher autoimmune prevalence. Asian race and BMI were inversely associated with autoimmune risk; higher income was unexpectedly associated with greater autoimmune diagnosis.

**Conclusions:**

Distinct sleep phenotypes were associated with autoimmune conditions. These associations may reflect shared or bidirectional links between sleep disruption and immune dysregulation.

## Highlights

• Sleep and circadian disorders were associated with autoimmune disease prevalence.
• DSPD, OSA, insomnia, and hypersomnia showed strong, non-causal associations.
• Autoimmune conditions were more prevalent among older adults and females.
• Asian race was associated with lower odds of autoimmune conditions.
• Findings may reflect bidirectional relationships between sleep and immune function.

## Graphical abstract

**Figure:**
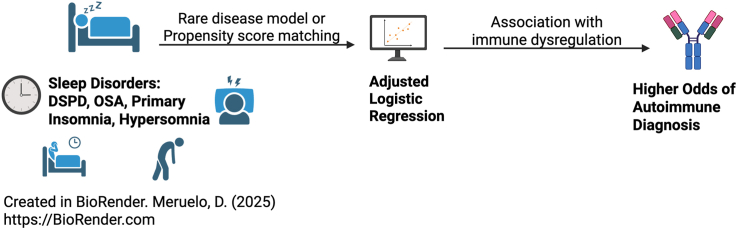

## 1 Introduction

Autoimmune diseases, which encompass a wide spectrum of chronic inflammatory conditions caused by dysregulation of the immune system (Arango et al., 2013), affect an estimated 5–10 % of the population (Conrad et al., 2023). There is growing evidence suggesting a rising prevalence and incidence of autoimmune diseases, posing a significant and increasing threat to global public health (Miller, 2023). Despite advances in understanding genetic susceptibility (Yasmeen et al., 2024) and environmental contributors (Agache et al., 2024), a substantial proportion of variance in autoimmune disease risk remains unexplained. In parallel, emerging research has highlighted the role of circadian biology and sleep health in immune regulation (Zeng et al., 2024), suggesting that disruptions in circadian rhythms or sleep architecture may contribute to immune dysfunction and inflammatory disease processes.

Sleep loss is associated with increases in inflammation and immune dysfunction that could relate to autoimmune disorders (Hsiao et al., 2015; Faraut et al., 2022). However, sleep is a complex phenotype reflecting the activities of the circadian clock, sleep pressure and arousal systems (Borbély, 2022) and it is not clear if the impact on immunity is similar across various kinds of sleep disruption arising from different causes. Presently, we examined four sleep and circadian disorders with distinctive etiologies to identify patterns of association with autoimmune disorders.

Circadian rhythms influence a range of immunological functions, including cytokine release, lymphocyte trafficking, and antigen presentation (Zeng et al., 2024). Experimental studies have demonstrated that circadian misalignment—such as that seen with irregular sleep schedules or nocturnal light exposure—can impair immunological function and amplify inflammatory responses (Zeng et al., 2024; Eckel et al., 2015; James et al., 2017). Chronotype (Roenneberg et al., 2019), an individual's natural preference for timing of sleep and activity, provides a behavioral marker of underlying circadian alignment or disruption and offers a real-world framework for studying its effects on health.

Delayed chronotype, often clinically diagnosed as delayed sleep phase disorder (DSPD) (Danielsson et al., 2016; Watson et al., 2018, 2020), is characterized by difficulty falling asleep and waking at socially conventional times. Individuals with delayed chronotype often experience chronic sleep deprivation, social jetlag, and metabolic dysregulation (Cespedes Feliciano et al., 2019), all of which have been independently associated with adverse health outcomes. Although delayed chronotype has been linked to higher rates of depression, anxiety (Taylor and Hasler, 2018; Harrison et al., 2021; Druiven et al., 2020), cardiometabolic disease (Cespedes Feliciano et al., 2019), and substance use (Hug et al., 2019; Kwon and Lee, 2022; Wittmann et al., 2010), its relationship to autoimmune disease remains poorly understood.

Other forms of sleep impairment such as obstructive sleep apnea (OSA) and insomnia may also contribute to immune dysfunction. OSA, characterized by intermittent airway collapse during sleep (Abbasi et al., 2021), results in increased arousal in response to hypoxia that causes sleep fragmentation, and is associated with systemic inflammation. Similarly, primary insomnia—marked by difficulty initiating or maintaining sleep occurring independently of other medical, psychiatric, or environmental causes—and hypersomnia—characterized by excessive daytime sleepiness—have been linked to dysregulated immune and inflammatory profiles (Garbarino et al., 2021). However, few large-scale studies have evaluated whether these common sleep disorders independently increase autoimmune disease risk.

To date, there have been no large, population-based studies systematically investigating whether distinct behavioral and physiological sleep phenotypes—such as DSPD, OSA, primary insomnia, or hypersomnia—are associated with increased autoimmune disease prevalence. Prior studies have largely focused on occupational circadian disruption, such as shift work, as a proxy for circadian misalignment (Faraut et al., 2022; Stenger et al., 2022; McCarthy, 2022), or have been restricted to small, homogenous clinical cohorts. Moreover, few studies have rigorously accounted for sociodemographic and clinical confounders that may influence both sleep and autoimmune risk, such as body mass index (BMI) (Balestrieri et al., 2020), income (Singh et al., 2017), chronic inflammatory conditions (Furman et al., 2019; Xiang et al., 2023), and smoking (Costenbader and Karlson 2006; Jaehne et al., 2012)—factors known to shape healthcare access, immune burden, and sleep regulation. The present study addresses these gaps by leveraging data from the All of Us Research Program The ‘All of Us’ Research Program, 2019)—a longitudinal, nationally representative cohort—to examine whether individuals with clinically diagnosed DSPD, OSA, primary insomnia, or hypersomnia show increased prevalence of autoimmune conditions compared to regular sleepers without these disorders.

We hypothesized that all four sleep disorders—DSPD, OSA, primary insomnia, and hypersomnia—would be independently associated with higher odds of autoimmune disease. Furthermore, we anticipated that these associations would remain significant after adjusting for key demographic and clinical covariates, including age, sex at birth, race, ethnicity, BMI, household income, chronic inflammatory diagnoses, and smoking history. By clarifying the relationships between multiple distinct forms of sleep disruption and immune health, this work aims to provide novel insight into circadian behavior, sleep physiology, and sleep pathology as potentially modifiable indicators of autoimmune disease susceptibility.

## 2 Methods

### 2.1 Data source

We utilized data from the All of Us Research Program Registered Tier Dataset (version 8) The ‘All of Us’ Research Program, 2019), a large, nationally representative, longitudinal cohort supported by the National Institutes of Health (NIH) that aims to accelerate biomedical research and improve health by gathering diverse health data from over one million participants across the United States. The dataset includes electronic health records (EHR), survey responses, physical measurements, and wearable device data. All analyses were conducted using R version 4.2.2 (R Core Team, 2021) within the secure, cloud-based All of Us Researcher Workbench environment.

### 2.2 Study population

This study included complementary analyses focusing on sleep-related phenotypes and autoimmune conditions. Four participant groups were defined based on full cohort data: individuals with a diagnosis of Delayed Sleep Phase Disorder (DSPD; n = 639), Obstructive Sleep Apnea (OSA; n = 13,493), Primary Insomnia (n = 1825), or Hypersomnia (n = 5502). Each group was compared to a corresponding set of regular sleepers without that sleep diagnosis (DSPD comparison: n = 424,456; OSA comparison: n = 138,851; Primary Insomnia comparison: n = 149,411; Hypersomnia comparison: n = 160,416). Each subgroup analysis was conducted independently, and sleep disorder groups were defined to be mutually exclusive. For each analysis, participants with the target sleep disorder were compared to a control group of regular sleepers, defined as individuals without any documented diagnosis of DSPD, OSA, Primary Insomnia, or Hypersomnia. Additionally, individuals with any of the other three sleep disorders were excluded from the target disorder group to ensure clear group separation. This approach eliminated diagnostic overlap across sleep disorder groups and enhanced the interpretability of between-group comparisons.

Autoimmune conditions were identified through structured electronic health record (EHR) diagnosis codes using a curated list of SNOMED identifiers. This list encompassed a broad range of autoimmune diseases, including systemic lupus erythematosus, multiple sclerosis, rheumatoid arthritis, and autoimmune thyroiditis. Diagnostic codes were selected based on existing literature (Arango et al., 2013; Conrad et al., 2023) and expert clinical review. Among individuals with autoimmune conditions (N = 69,452), the most common diagnoses were Rheumatoid Arthritis (16.4 %), Psoriasis (15.3 %), Type 1 Diabetes Mellitus (11.7 %), Systemic Lupus Erythematosus (7.2 %), Crohn's Disease (6.1 %), and Ulcerative Colitis (5.9 %). Less frequent conditions, including Hashimoto's Thyroiditis, Graves' Disease, and Celiac Disease, were grouped under ‘Other’ due to lower prevalence (<5 % each).

### 2.3 Variables

Sleep phenotypes were defined based on clinical EHR diagnoses. Participants with Delayed Sleep Phase Disorder (DSPD), Obstructive Sleep Apnea (OSA), Primary Insomnia, or Hypersomnia were assigned to their respective sleep disorder group for each analysis. Regular sleepers were defined as individuals without any diagnosis of the four specified sleep disorders. The outcome variable was autoimmune disease status, defined as a binary indicator of whether the participant had one or more qualifying autoimmune conditions.

Covariates included age (continuous, in years), sex at birth, race, ethnicity, household income, BMI, and a binary indicator of chronic inflammatory disease burden. Sex at birth, race, and ethnicity were defined based on All of Us survey responses. Race was coded into the following categories: White, Black or African American, Asian, More than one population, Another single population, None of these, I prefer not to answer, None indicated, and PMI: Skip. Ethnicity was categorized as Hispanic or Latino, Not Hispanic or Latino, Prefer Not to Answer, PMI: Skip, or Race/Ethnicity None of These. Household income was treated as a categorical variable with levels for: <$10,000, $10,000–$24,999, $25,000–$49,999, $50,000–$99,999, ≥$100,000, and “Prefer not to answer.” BMI was computed from the most recent available EHR-recorded height and weight values.

The chronic inflammatory diagnosis variable was defined as a binary indicator reflecting whether a participant had at least one documented EHR diagnosis corresponding to a predefined set of chronic inflammatory conditions. These conditions were selected based on their established role in systemic inflammation and their inclusion in the Charlson Comorbidity Index (Charlson et al., 1987). SNOMED concept sets were constructed using the following standard concept IDs: 13645005 (Chronic obstructive lung disease), 195951007 (Acute exacerbation of chronic obstructive airways disease), 196001008 (Chronic obstructive pulmonary disease with acute lower respiratory infection), 10692761000119107 (Asthma-chronic obstructive pulmonary disease overlap syndrome), 709044004 (Chronic kidney disease), 328383001 (Chronic liver disease), 44054006 (Type 2 diabetes mellitus), 313436004 (Type 2 diabetes mellitus without complication), 368051000119109 (Hyperglycemia due to type 2 diabetes mellitus), 42343007 (Congestive heart failure), 88805009 (Chronic congestive heart failure), 400047006 (Peripheral vascular disease), 13200003 (Peptic ulcer), 22298006 (Myocardial infarction), and 62914000 (Cerebrovascular disease). Participants with at least one diagnosis from this list were coded as positive for chronic inflammatory burden. Concept sets were defined using standard SNOMED logic within the All of Us Researcher Workbench.

Ambiguous or non-informative responses to survey questions, such as “PMI Skip,” “Don't Know,” or “Prefer not to answer,” were retained as explicit categories in all models to ensure transparency and were examined in sensitivity analyses described below.

### 2.4 Statistical analysis

Descriptive analyses compared demographic and clinical characteristics between individuals with and without autoimmune disease. Continuous variables were summarized using means and standard deviations, while categorical variables were reported as counts and percentages. Between-group comparisons were conducted using Welch's *t*-tests for continuous variables and chi-squared or Fisher's exact tests for categorical variables, depending on cell sizes.

To complement *p*-values, effect sizes were calculated to quantify the magnitude of group differences. Cohen's *d* was used for continuous variables, and Cramér's *V* was used for categorical variables. Effect sizes were interpreted according to established guidelines, with thresholds for small (*d* = 0.2; *V* = 0.1), medium (*d* = 0.5; *V* = 0.3), and large (*d* = 0.8; *V* = 0.5) effects (Cohen, 1988).

Logistic regression models were used to assess whether each sleep phenotype—Delayed Sleep Phase Disorder (DSPD), Obstructive Sleep Apnea (OSA), Primary Insomnia, and Hypersomnia—was associated with odds of autoimmune disease. Two complementary analytic approaches were used. For DSPD and Primary Insomnia, a rare disease case-control design was employed (Tenny et al. 2025), in which all autoimmune cases were retained and a 5:1 random sample of controls without autoimmune disease was selected to mitigate class imbalance and reduce computational burden. For OSA and Hypersomnia, we applied 1:5 propensity score matching without replacement using nearest-neighbor algorithms, with covariates including age, sex at birth, race, ethnicity, BMI, and income level. These post-matching subsets were used for all regression modeling, while descriptive comparisons (e.g., Table 1, Table 2, Table 3, Table 4) were based on the full sample.

**Table 1:** Demographic comparison between individuals with and without autoimmune conditions (delayed sleep phase disorder).

Variable	No	Yes	p-value	Effect Size
Group: DSPD ∗	29 (0.0 %)	30 (0.1 %)	6.36e-12	0.02
Group: Regular	147806 (100.0 %)	29537 (99.9 %)		
Age (Mean ± SD) ∗	52.7 ± 16.6	58.3 ± 16.0	0	−0.35
Sex Assigned at Birth: Female ∗	93339 (63.1 %)	21014 (71.1 %)	2.98e-153	0.06
Sex Assigned at Birth: Male	53029 (35.9 %)	8228 (27.8 %)		
Sex: No Matching Concept	57 (0.0 %)	–		
Sex: Other/Skipped/Prefer Not to Answer	1410 (1.0 %)	305 (1.0 %)		
Race: White ∗	79027 (53.5 %)	18084 (61.2 %)	2.18e-159	0.07
Race: Another Single Population	3318 (2.2 %)	557 (1.9 %)		
Race: Asian	5995 (4.1 %)	610 (2.1 %)		
Race: Black or African American	24546 (16.6 %)	4119 (13.9 %)		
Race: I Prefer Not to Answer	816 (0.6 %)	147 (0.5 %)		
Race: More Than One Population	7511 (5.1 %)	1333 (4.5 %)		
Race: None Indicated	23396 (15.8 %)	4010 (13.6 %)		
Race: None of These	1621 (1.1 %)	323 (1.1 %)		
Race: PMI Skip	1605 (1.1 %)	384 (1.3 %)		
Ethnicity: Hispanic or Latino ∗	28919 (19.6 %)	4901 (16.6 %)	4.42e-31	0.03
Ethnicity: No Matching Concept	–	0 (0.0 %)		
Ethnicity: Not Hispanic or Latino	114872 (77.7 %)	23812 (80.5 %)		
Ethnicity: Prefer Not to Answer	816 (0.6 %)	147 (0.5 %)		
Ethnicity: PMI Skip	1605 (1.1 %)	384 (1.3 %)		
Ethnicity: None of These	1621 (1.1 %)	323 (1.1 %)		

This table summarizes demographic characteristics for individuals with and without autoimmune conditions in the matched DSPD rare disease sample (N ≈ 151,000). Categorical variables are presented as counts (percentages), and continuous variables as mean ± standard deviation. P-values were calculated using chi-squared tests for categorical variables and Welch's *t*-test for continuous variables, with significant p-values shown. Effect sizes are reported as Cramér's V for categorical comparisons and Cohen's d for continuous comparisons. “--” indicates suppressed counts per All of Us reporting guidelines (<20 participants). Asterisks (∗) denote statistical significance at p < 0.05.

**Table 2:** Demographic comparison between individuals with and without autoimmune conditions (obstructive sleep apnea).

Variable	No	Yes	p-value	Effect Size
Group: OSA ∗	26,318 (7.7 %)	6300 (21.7 %)	0	0.13
Group: Regular	314,758 (92.3 %)	22,683 (78.3 %)		
Age (Mean ± SD) ∗	55.5 ± 16.8	60.1 ± 15.4	0	−0.28
Sex Assigned at Birth: Female ∗	203,749 (59.7 %)	19,833 (68.4 %)	3.95e-189	0.05
Sex Assigned at Birth: Male	134,455 (39.4 %)	8873 (30.6 %)		
Sex: No Matching Concept	131 (0.0 %)	–		
Sex: Other/Skipped/Prefer Not to Answer	2741 (0.8 %)	261 (0.9 %)		
Race: White ∗	197,924 (58.0 %)	19,022 (65.6 %)	1.61e-176	0.05
Race: Another Single Population	6687 (2.0 %)	450 (1.6 %)		
Race: Asian	11,994 (3.5 %)	523 (1.8 %)		
Race: Black or African American	56,905 (16.7 %)	3944 (13.6 %)		
Race: I Prefer Not to Answer	1308 (0.4 %)	107 (0.4 %)		
Race: More Than One Population	16,067 (4.7 %)	1361 (4.7 %)		
Race: None Indicated	43,569 (12.8 %)	2919 (10.1 %)		
Race: None of These	3280 (1.0 %)	314 (1.1 %)		
Race: PMI Skip	3342 (1.0 %)	343 (1.2 %)		
Ethnicity: Hispanic or Latino ∗	54,922 (16.1 %)	3721 (12.8 %)	3.47e-47	0.02
Ethnicity: Not Hispanic or Latino	278,224 (81.6 %)	24,498 (84.5 %)		
Ethnicity: Prefer Not to Answer	1308 (0.4 %)	107 (0.4 %)		
Ethnicity: PMI Skip	3342 (1.0 %)	343 (1.2 %)		
Ethnicity: None of These	3280 (1.0 %)	314 (1.1 %)		

This table summarizes demographic characteristics for individuals with and without autoimmune conditions in the matched OSA propensity score–matched sample (N ≈ 373,000). Categorical variables are presented as counts (percentages), and continuous variables as mean ± standard deviation. P-values were calculated using chi-squared tests for categorical variables and Welch's *t*-test for continuous variables. Effect sizes are reported as Cramér's V for categorical comparisons and Cohen's d for continuous comparisons. “--” indicates suppressed counts per All of Us reporting guidelines (<20 participants). Asterisks (∗) denote statistical significance at p < 0.05.

**Table 3:** Demographic comparison between individuals with and without autoimmune conditions (primary insomnia).

Variable	No	Yes	p-value	Effect Size
Group: Primary Insomnia ∗	1086 (0.7 %)	788 (2.6 %)	1.26e-192	0.07
Group: Regular	150,539 (99.3 %)	29,537 (97.4 %)		
Age (Mean ± SD) ∗	52.8 ± 16.6	58.5 ± 16.0	0	−0.35
Sex Assigned at Birth: Female ∗	95,811 (63.2 %)	21,591 (71.2 %)	2.45e-160	0.06
Sex Assigned at Birth: Male	54,314 (35.8 %)	8400 (27.7 %)		
Sex: No Matching Concept	58 (0.0 %)	–		
Sex: Other/Skipped/Prefer Not to Answer	1442 (1.0 %)	314 (1.0 %)		
Race: White ∗	81,167 (53.5 %)	18,581 (61.3 %)	3.23e-162	0.07
Race: Another Single Population	3403 (2.2 %)	569 (1.9 %)		
Race: Asian	6069 (4.0 %)	628 (2.1 %)		
Race: Black or African American	25,177 (16.6 %)	4218 (13.9 %)		
Race: I Prefer Not to Answer	838 (0.6 %)	151 (0.5 %)		
Race: More Than One Population	7694 (5.1 %)	1366 (4.5 %)		
Race: None Indicated	23,955 (15.8 %)	4083 (13.5 %)		
Race: None of These	1603 (1.1 %)	333 (1.1 %)		
Race: PMI Skip	1719 (1.1 %)	396 (1.3 %)		
Ethnicity: Not Hispanic or Latino	117,871 (77.7 %)	24,455 (80.6 %)	–	–
Ethnicity: Hispanic or Latino	29,594 (19.5 %)	4990 (16.5 %)		
Ethnicity: No Matching Concept	0 (0.0 %)	0 (0.0 %)		
Ethnicity: Prefer Not to Answer	838 (0.6 %)	151 (0.5 %)		
Ethnicity: PMI Skip	1719 (1.1 %)	396 (1.3 %)		
Ethnicity: None of These	1603 (1.1 %)	333 (1.1 %)		

This table summarizes demographic characteristics for individuals with and without autoimmune conditions in the Primary Insomnia rare disease sample (N ≈ 182,000). Categorical variables are presented as counts (percentages), and continuous variables as mean ± standard deviation. P-values were calculated using chi-squared tests for categorical variables and Welch's *t*-test for continuous variables, with significant p-values shown. Effect sizes are reported as Cramér's V for categorical comparisons and Cohen's d for continuous comparisons. “--” indicates suppressed counts per All of Us reporting guidelines (<20 participants). P-value and effect size for ethnicity were not calculated due to small cell counts. Asterisks (∗) denote statistical significance at p < 0.05.

**Table 4:** Demographic comparison between individuals with and without hypersomnia.

Variable	Regular Group (No)	Hypersomnia Group (Yes)	p-value	Effect Size
Group: Hypersomnia ∗	2837 (0.9 %)	765 (3.3 %)	1.45e-256	0.06
Group: Regular	314,758 (99.1 %)	22,683 (96.7 %)		
Age (Mean ± SD) ∗	54.8 ± 16.9	58.8 ± 15.8	3.79e-285	−0.24
Sex Assigned at Birth: Female ∗	193,089 (60.8 %)	16,652 (71.0 %)	5.64e-215	0.05
Sex Assigned at Birth: Male	121,868 (38.4 %)	6576 (28.0 %)		
Sex: No Matching Concept	120 (0.0 %)	–		
Sex: Other/Skipped/Prefer Not to Answer	2518 (0.8 %)	207 (0.9 %)		
Race: White ∗	182,183 (57.4 %)	15,373 (65.6 %)	1.31e-159	0.05
Race: Another Single Population	6346 (2.0 %)	370 (1.6 %)		
Race: Asian	11,657 (3.7 %)	455 (1.9 %)		
Race: Black or African American	53,443 (16.8 %)	3118 (13.3 %)		
Race: I Prefer Not to Answer	1204 (0.4 %)	83 (0.4 %)		
Race: More Than One Population	14,989 (4.7 %)	1093 (4.7 %)		
Race: None Indicated	41,661 (13.1 %)	2427 (10.4 %)		
Race: None of These	3068 (1.0 %)	257 (1.1 %)		
Race: PMI Skip	3044 (1.0 %)	272 (1.2 %)		
Ethnicity: Not Hispanic or Latino ∗	257,865 (81.2 %)	19,727 (84.1 %)	2.44e-37	0.02
Ethnicity: Hispanic or Latino	52,414 (16.5 %)	3109 (13.3 %)		
Ethnicity: No Matching Concept	–	–		
Ethnicity: Prefer Not to Answer	1204 (0.4 %)	83 (0.4 %)		
Ethnicity: PMI Skip	3044 (1.0 %)	272 (1.2 %)		
Ethnicity: None of These	3068 (1.0 %)	257 (1.1 %)		

This table summarizes demographic characteristics for individuals with and without hypersomnia in the matched sample (N = 320,043). Categorical variables are presented as counts (percentages), and continuous variables as mean ± standard deviation. P-values were calculated using chi-squared tests for categorical variables and Welch's t-tests for continuous variables, with significant p-values shown. Effect sizes are reported as Cramér's V for categorical comparisons and Cohen's d for continuous comparisons. “--” indicates suppressed counts per All of Us reporting guidelines (<20 participants). Asterisks (∗) denote statistical significance at p < 0.05.

Models compared individuals with regular sleep patterns to those with the respective sleep disorder, with the sleep disorder group specified as the reference category. Thus, odds ratios less than 1 indicate reduced odds of autoimmune disease among regular sleepers relative to those with the sleep disorder.

All regression models were adjusted for age, sex at birth, race, ethnicity, household income, BMI, and presence of a chronic inflammatory diagnosis (Sperandei, 2014). The reference group for race was “White”; this group was selected due to its sufficient size and stability. Interaction terms were excluded due to quasi-complete separation in preliminary models. Adjusted odds ratios (ORs), 95 % confidence intervals (CIs), and p-values were reported. ORs above 1 indicated increased odds of autoimmune disease, while ORs below 1 indicated reduced odds (Chen et al., 2010). Statistical significance was determined by whether the 95 % CIs excluded 1.0 (Hazra, 2017) and further confirmed using false discovery rate (FDR) correction via the Benjamini-Hochberg procedure (Benjamini and Hochberg, 1995), with adjusted p-values less than 0.05 considered significant.

### 2.5 Propensity score matching and covariate balance

We constructed four analytic samples corresponding to sleep-related phenotypes: DSPD, OSA, Primary Insomnia, and Hypersomnia. For each group, individuals with autoimmune conditions (“cases”) were identified. For the DSPD and Primary Insomnia models, we used a rare disease case-control sampling strategy by selecting five controls without autoimmune disease for each case. These samples were not matched but were analyzed using logistic regression models adjusting for age, sex at birth, race, ethnicity, household income, BMI, and chronic inflammatory conditions.

For the OSA and Hypersomnia models, we applied 1:5 nearest-neighbor propensity score matching with replacement using the MatchIt R package (Ho et al., .). Propensity scores were estimated via logistic regression using the same set of covariates: age, sex at birth, race, ethnicity, household income, BMI, and chronic inflammatory conditions. Matching with replacement was used to improve match quality, particularly where the pool of eligible controls was limited or dissimilar to the cases. Because matching with replacement results in repeated use of some control participants and violates the assumption of independent observations, cluster-robust standard errors (Cameron and Miller, 2015) were calculated in all post-matching regression models, clustering on participant ID to account for this non-independence.

Covariate balance was assessed before and after matching using standardized mean differences and visualized using Love plots generated with the cobalt package (“Introduction, .; Zhang et al., 2019). Demographic characteristics for each analytic sample are summarized in Table 1, Table 2, Table 3, Table 4.

### 2.6 Sensitivity analyses

To assess robustness to unmeasured confounding, E-value analyses (Chung and Chung 2023) were conducted separately for each sleep phenotype model. E-values quantify the minimum strength of association that an unmeasured confounder would need to have with both the exposure and outcome, conditional on measured covariates, to fully explain away the observed association.

Sensitivity analyses were tailored to the modeling strategy used for each sleep phenotype. For the Delayed Sleep Phase Disorder (DSPD) and Primary Insomnia models, which used a rare disease case-control approach with a 1:5 ratio of cases to unmatched controls, sensitivity checks included: (1) excluding participants with ambiguous demographic codes across sex, race, and ethnicity (e.g., “PMI Skip,” “Prefer not to answer,” and “No matching concept”); (2) altering reference categories to “Regular” sleepers, “Male” sex, and “Black or African American” race; (3) restricting the sample to complete cases for income and BMI; and (4) adjusting for self-reported smoking frequency among participants with non-missing data. For the Obstructive Sleep Apnea (OSA) and Hypersomnia models, which were based on 1:5 propensity score–matched samples, the same sensitivity analyses were applied using the matched datasets and original covariate set.

## 3 Results

### 3.1 Prevalence of autoimmune conditions by sleep disorder group

Individuals diagnosed with delayed sleep phase disorder (DSPD) exhibited a markedly higher prevalence of autoimmune conditions (0.6 %) compared to regular sleepers (0.1 %) (Table 1). A Fisher's exact test confirmed this difference was statistically significant (p = 6.36e-12). The unadjusted odds ratio (OR) was 0.194 (95 % CI: 0.155–0.244), indicating that regular sleepers had substantially lower odds of having an autoimmune condition relative to those with DSPD.

Similarly, individuals with obstructive sleep apnea (OSA) demonstrated a significantly higher prevalence of autoimmune conditions compared to individuals without OSA (21.7 % vs 7.7 %; Table 2; p < 2.2e-16), with an unadjusted OR of 0.229 (95 % CI: 0.221–0.238). In the Primary Insomnia group, 2.6 % had an autoimmune diagnosis compared to 0.7 % of regular sleepers (Table 3; p = 1.26e-192), with an unadjusted OR of 0.294 (95 % CI: 0.267–0.323). Among individuals with Hypersomnia, 3.3 % had an autoimmune condition compared to 0.9 % of regular sleepers (Table 4; p = 1.45e-256), with an unadjusted OR of 0.256 (95 % CI: 0.245–0.269).

### 3.2 Propensity score matching diagnostics

Propensity score matching was applied to the OSA and Hypersomnia groups to facilitate covariate-balanced comparisons with regular sleepers. After matching on age, sex at birth, race, and ethnicity using a 1:5 nearest neighbor approach without replacement, covariate balance was assessed using standardized mean differences and visualized in Supplementary Figs. 1 and 2. In both matched datasets, all covariates achieved satisfactory balance, with absolute standardized mean differences well below the conventional 0.1 threshold, indicating that the matching procedure was effective in minimizing confounding by measured demographic variables. No meaningful imbalance remained between comparison groups following matching.

### 3.3 Adjusted logistic regression

**Fig. 1:**
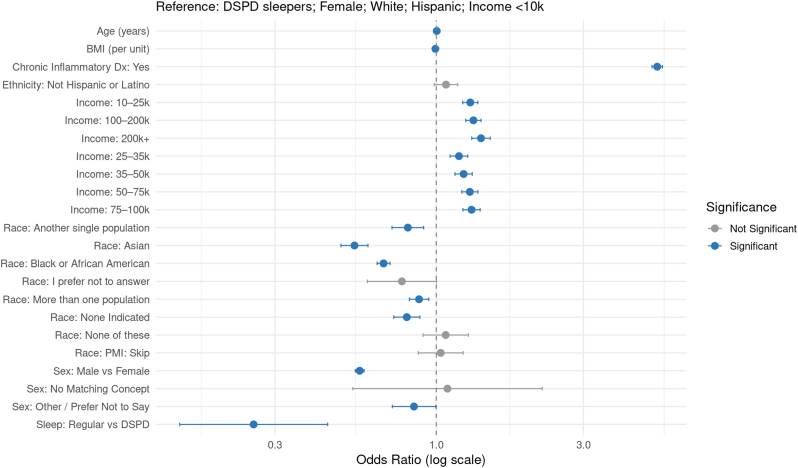
Adjusted Odds Ratios for Autoimmune Diagnosis Following Propensity Score Matching (Delayed Sleep Phase Disorder) Forest plot displaying adjusted odds ratios (ORs) and 95 % confidence intervals (CIs) for the association between delayed sleep phase disorder (DSPD) and autoimmune diagnosis, based on a rare disease case-control design with 1:5 matching of controls to cases. Logistic regression with robust standard errors (clustered by participant) was used to estimate associations, adjusting for demographic and clinical covariates. Odds ratios are plotted on a logarithmic scale. Blue points indicate statistically significant associations (FDR-adjusted p < 0.05); gray points indicate non-significant associations. Reference groups: Sleep = DSPD, Sex = Female, Race = White, Ethnicity = Hispanic or Latino, Income = <10k.

In the DSPD model (Supplementary Table 1; Fig. 1), regular sleepers had significantly lower odds of autoimmune disease compared to individuals with DSPD after covariate adjustment. The adjusted OR was 0.256 (95 % CI: 0.147–0.445; p = 2.3e-04), consistent with the unadjusted findings. The E-value for the point estimate was 7.27, and for the lower CI bound was 13.04, indicating substantial robustness to unmeasured confounding.

**Fig. 2:**
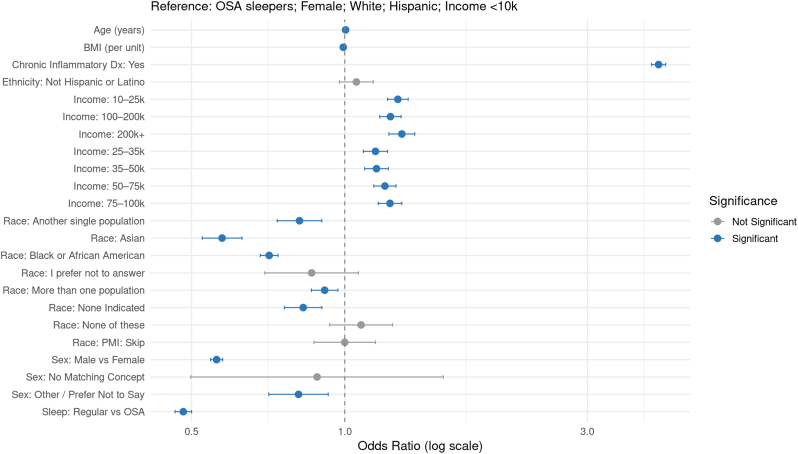
Adjusted Odds Ratios for Autoimmune Diagnosis Following Propensity Score Matching (Obstructive Sleep Apnea) Forest plot showing adjusted odds ratios (ORs) and 95 % confidence intervals (CIs) for the association between OSA status and autoimmune disease diagnosis, as well as demographic covariates, following 1:5 case-control propensity score matching. Odds ratios are plotted on a log scale. Robust standard errors were used to account for potential clustering and heteroskedasticity in the matched sample. Blue points indicate statistically significant associations (FDR-adjusted p < 0.05), and gray points indicate non-significant associations. Reference groups: Sleep = OSA, Sex = Female, Race = White, and Ethnicity = Hispanic.

In the OSA model (Supplementary Table 2; Fig. 2), which followed covariate-matched analysis, individuals without OSA had substantially lower odds of autoimmune disease compared to those with OSA. The adjusted OR was 0.461 (95 % CI: 0.411–0.517; p < 2e-16). E-values for the point estimate and CI lower bound were 3.57 and 3.73, respectively, supporting a moderate degree of resistance to unmeasured confounding.

**Fig. 3:**
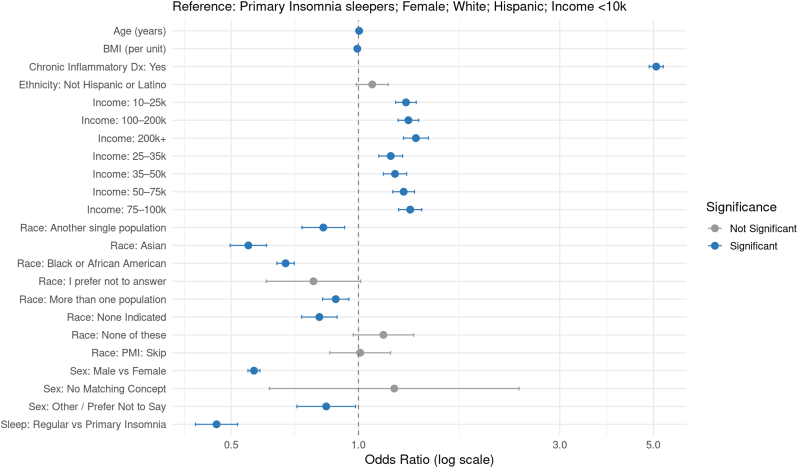
Adjusted Odds Ratios for Autoimmune Diagnosis (Primary Insomnia) Forest plot showing adjusted odds ratios (ORs) and 95 % confidence intervals (CIs) for the association between sleep group (Regular vs Primary Insomnia) and autoimmune disease diagnosis, as well as demographic covariates, using a rare disease logistic regression model. Odds ratios are plotted on a log scale. Blue points indicate statistically significant associations (FDR-adjusted *p* < 0.05), while gray points indicate non-significant associations. Reference groups: Sleep = Primary Insomnia, Sex = Female, Race = White, Ethnicity = Hispanic, and Income = <10k.

In the Primary Insomnia model (Supplementary Table 3; Fig. 3), which also used the rare disease design, regular sleepers again had significantly lower odds of autoimmune conditions compared to those diagnosed with Primary Insomnia (adjusted OR = 0.461; 95 % CI: 0.411–0.517; p < 2e-16). The E-value for the point estimate was 3.76, and 4.30 for the lower confidence bound.

**Fig. 4:**
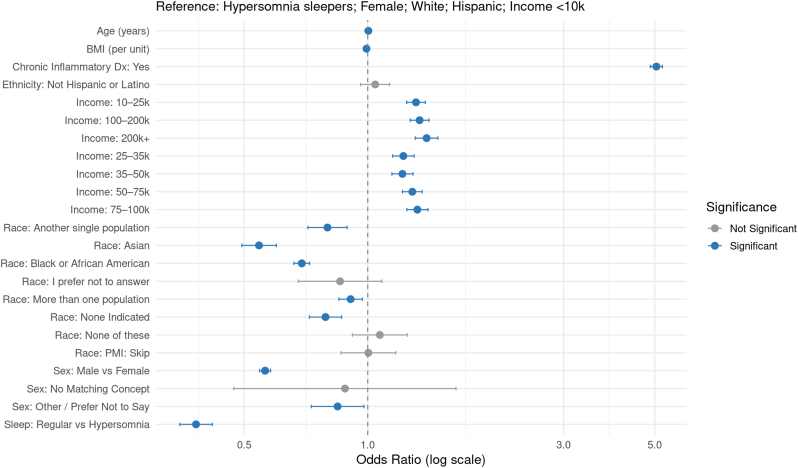
Adjusted Odds Ratios for Autoimmune Diagnosis Following Propensity Score Matching (Hypersomnia) Forest plot showing adjusted odds ratios (ORs) and 95 % confidence intervals (CIs) for predictors of autoimmune disease diagnosis in a propensity score–matched sample (1:5 matching). Odds ratios are displayed on a logarithmic scale. Blue points indicate statistically significant associations (FDR-adjusted *p* < 0.05); gray points indicate non-significant associations. Reference groups: Sleep = Hypersomnia, Sex = Female, Race = White, Ethnicity = Hispanic, and Income < $10,000.

In the Hypersomnia model (Supplementary Table 4; Fig. 4), propensity score-matched comparisons showed that individuals without Hypersomnia had significantly lower odds of autoimmune diagnosis (adjusted OR = 0.482; 95 % CI: 0.464–0.500; p < 2e-16). Corresponding E-values were 4.68 and 5.18.

### 3.4 Covariate effects across models

Across all four models, increasing age was significantly associated with autoimmune diagnosis (OR per year ≈ 1.00; all p < 1e-09), though with a small effect size. Male sex conferred a protective effect across all sleep disorder groups (e.g., DSPD model: OR = 0.565; OSA model: OR = 0.566; Primary Insomnia model: OR = 0.566; Hypersomnia model: OR = 0.560; all p < 2e-16). Chronic inflammatory diagnosis was the strongest predictor of autoimmune status, with adjusted ORs between 4.14 and 5.20 across models (all p < 2e-16).

Racial and ethnic differences were consistent. Asian individuals had significantly lower odds of autoimmune disease compared to the reference group (“Another single population”) across all models (OR range: 0.543–0.574; all p < 2e-16). Black or African American individuals similarly had lower odds across all groups (OR range: 0.672–0.710). White individuals showed elevated odds relative to the reference group (OR ∼1.17–1.19), although White was not explicitly reported due to reference coding. Other racial categories such as “More than one population,” “None indicated,” and “I prefer not to answer” showed weaker or inconsistent associations.

Household income was positively associated with autoimmune diagnosis in all models. Individuals earning above $10,000 per year had significantly greater odds of autoimmune disease than those in the lowest income bracket (<$10k), with ORs ranging from 1.15 to 1.40 (all FDR-adjusted p < 1e-04). BMI was inversely associated with autoimmune status (OR range: 0.993–0.994 per unit; all p < 1e-05), likely due to statistical adjustment for chronic inflammatory conditions. Ethnicity (Not Hispanic or Latino vs. Hispanic or Latino) was not significantly associated with autoimmune risk in any model (all adjusted p > 0.05).

### 3.5 Sensitivity analysis

E-value analysis was used to evaluate the potential impact of unmeasured confounding. In the DSPD model, an unmeasured confounder would need a relative risk of at least 7.27 with both chronotype and autoimmune diagnosis to explain the association, and 13.04 to account for the lower bound of the confidence interval. Comparable E-values were observed in other models: 3.57 (CI: 3.73) for OSA, 4.68 (CI: 5.18) for Hypersomnia, and 3.76 (CI: 4.30) for Primary Insomnia. These values suggest that substantial unmeasured confounding would be required to fully explain away the observed associations.

Additional sensitivity analyses tested the robustness of results across alternative model specifications: (1) excluding ambiguous demographic responses (e.g., “PMI Skip,” “Prefer not to answer”), (2) altering reference categories for sex, race, and sleep group, (3) restricting to complete income and BMI data, and (4) adding smoking frequency as a covariate. In all cases, associations between sleep phenotypes and autoimmune diagnoses remained statistically significant and directionally consistent, reinforcing the validity of the primary findings.

## 4 Discussion

In this large, population-based study using data from the All of Us Research The ‘All of Us’ Research Program, 2019), we found that individuals with delayed sleep phase disorder (DSPD), obstructive sleep apnea (OSA), primary insomnia, or hypersomnia had significantly higher odds of autoimmune disease diagnoses compared to regular sleepers. These associations persisted after rigorous covariate adjustment, including age, sex, race, ethnicity, income, BMI, and chronic inflammatory burden. While DSPD and primary insomnia were analyzed using a rare disease logistic regression approach, OSA and hypersomnia groups underwent propensity score matching to ensure covariate balance. Despite these analytic differences, we observed consistent effect sizes across models, with adjusted odds ratios ranging from 0.256 to 0.482. These findings extend prior clinical and epidemiologic research demonstrating links between sleep disturbance and immune dysfunction (Zeng et al., 2024; Faraut et al., 2022; Stenger et al., 2022; McCarthy, 2022). While earlier studies focused on shift work as a model of circadian misalignment (James et al., 2017; Faraut et al., 2022; Stenger et al., 2022), our results show that trait-like sleep phenotypes—including altered sleep timing, disordered breathing, insomnia symptoms, and hypersomnia—are also associated with increased autoimmune disease risk. Our work builds upon and replicates the findings of (Hsiao et al., 2015), who reported associations between non-apnea sleep disorders and autoimmune conditions in a Taiwanese cohort. By using a large and demographically diverse U.S. sample, our study strengthens the evidence base and underscores the reproducibility of these associations across populations and diagnostic categories.

Mechanistically, distinct sleep phenotypes may reflect partially overlapping immune pathways. Delayed sleep timing and insomnia may impair the circadian regulation of immunologic function, thereby increasing pro-inflammatory signaling. Hypersomnia may reflect a compensatory response to systemic immune activation, as excessive sleepiness has been linked to chronic inflammation in prior studies (Schneider et al., 2025). Obstructive sleep apnea, in contrast, may promote immune dysregulation through hypoxia-induced cytokine release, glucocorticoid rhythm disruption, and sympathetic overactivation (Lin et al., 2024; Ding et al., 2024; Zeng et al., 2024). Although directionality remains uncertain, the consistent strength of association across these sleep disorders supports the broader hypothesis that both biologically and behaviorally mediated sleep disturbances may co-occur with immune dysfunction.

In addition to sleep phenotypes, several covariates emerged as independent predictors of autoimmune diagnosis. Individuals with a history of chronic inflammatory diagnoses exhibited more than fourfold greater odds of autoimmune disease across all models, underscoring the interconnectedness of inflammation and immune regulation (Xiang et al., 2023; Furman et al., 2019). Higher income was paradoxically associated with increased odds of autoimmune diagnoses, with all income brackets above $10,000 showing significantly elevated risk. One plausible explanation is that individuals in the lowest income bracket (<$10k)—the reference group—may be underdiagnosed due to lack of access to routine healthcare and specialist evaluation (Singh et al., 2017). Similarly, BMI showed a small but statistically significant inverse association with autoimmune diagnosis, possibly reflecting residual confounding by health-seeking behavior or clinical status, as weight loss can accompany chronic inflammatory disease (Balestrieri et al., 2020). These findings suggest that clinical, behavioral, and socioeconomic factors may all interact in shaping autoimmune risk and detection.

We also replicated previously observed demographic associations with autoimmunity. Across all models, female sex and older age were associated with higher odds of autoimmune diagnosis, consistent with established sex- and age-related differences in immune function and autoimmunity (Desai and Brinton, 2019; Ngo et al., 2014; Kronzer et al., 2020; Angum et al., .; Bryl and Witkowski, 2019; Ramos-Casals et al., 2004; Zheng et al., 2023). Asian-identifying participants consistently showed lower odds of autoimmune diagnosis, while those identifying as White exhibited elevated risk across models. After adjusting for other variables, ethnicity (Hispanic vs. non-Hispanic) was not a significant predictor, suggesting that racial patterns may reflect more salient biological or structural disparities than ethnicity alone.

Despite the strengths of a large, nationally representative cohort and robust statistical modeling strategies, several limitations should be acknowledged. First, the cross-sectional nature of the data precludes any conclusions about temporal ordering or causality. It remains unclear whether sleep disruption increases autoimmune risk, whether autoimmune processes disturb sleep, or whether both are influenced by shared underlying factors. Although we interpret our findings cautiously, we emphasize that the associations may be bidirectional and warrant longitudinal investigation. Second, reliance on EHR-coded diagnoses may result in underdiagnosis or misclassification, especially for participants with limited healthcare access or milder disease presentations. Third, although we adjusted for a broad range of covariates, residual confounding is possible. While smoking was examined in sensitivity analyses and did not meaningfully alter the primary results, it was unexpectedly associated with slightly lower odds of autoimmune diagnosis—an observation that may reflect reporting bias, survival effects, or selection bias. However, other potential confounders, such as medication use (e.g., corticosteroids or immunosuppressants), could not be included due to insufficient data coverage, limiting our ability to account for their effects. Additionally, co-occurring medical conditions unrelated to sleep or immune health may have influenced the observed associations.

Future work should prioritize longitudinal designs with repeated measures of sleep behavior (e.g., actigraphy, polysomnography), biomarkers of inflammation, and detailed autoimmune phenotyping to clarify directionality and mechanistic pathways. Incorporating medication use, health access variables, and genetic risk scores will also enhance model precision. Clinical trials aimed at improving sleep timing (e.g., melatonin, chronotherapy), breathing (e.g., CPAP for OSA), and behavioral regulation (e.g., CBT-I) could help determine whether sleep-focused interventions reduce autoimmune symptoms or incidence.

In conclusion, we find that DSPD, OSA, primary insomnia, and hypersomnia are all independently associated with higher prevalence of autoimmune disease. These associations persisted even after adjusting for demographic, clinical, and socioeconomic factors, including chronic inflammatory conditions, BMI, income, and—based on sensitivity analysis—smoking. While our cross-sectional design cannot determine causality, the results support the role of sleep phenotypes as potential clinical indicators of immune dysregulation. Recognizing these sleep disorders in routine care may help identify individuals at risk for autoimmune disease and inform future prevention or intervention strategies focused on sleep health.

## CRediT authorship contribution statement

**Amber R. Li:** Writing – review & editing, Formal analysis. **Bhaavyaa Shah:** Writing – review & editing. **Michael L. Thomas:** Writing – review & editing, Methodology. **Michael J. McCarthy:** Writing – review & editing, Methodology, Funding acquisition. **Alejandro D. Meruelo:** Writing – review & editing, Writing – original draft, Visualization, Supervision, Methodology, Funding acquisition, Formal analysis, Conceptualization.

## Ethics approval and consent to participate

This study utilized data from the All of Us Research Program, where all participants provided informed consent for the collection and use of their health information. Analyses were conducted in accordance with the Declaration of Helsinki and approved through the All of Us data access and governance process. Institutional Review Board (IRB) oversight for the All of Us Research Program is managed centrally by the All of Us IRB.

## Data Statement

The data used in this study are available to qualified researchers through the All of Us Research Hub (https://www.researchallofus.org/), following approval and in compliance with the program's data access protocols. Researchers must be registered users and complete required training and data use agreements to access the de-identified datasets.

## Funding sources

This research was supported by the 10.13039/100000027National Institute on Alcohol Abuse and Alcoholism (10.13039/100000027NIAAA) under grant K23 AA026869 (Principal Investigator: Alejandro D. Meruelo, MD, PhD).

Additional support was provided by a VA Merit Award (BX003431) awarded to Michael J. McCarthy, MD, PhD.

## Declaration of competing interest

The authors declare no conflicts of interest beyond the research funding disclosed below from the 10.13039/100000027National Institute on Alcohol Abuse and Alcoholism and the U.S. Department of Veterans Affairs.

## Data Availability

Please see Data Statement.
