# Supplementary material for: Sleep and circadian disorders as risk factors for autoimmune disease: A population-based study
Source: Neurobiol Sleep Circadian Rhythms. 2025 May 20;18:100129. doi: 10.1016/j.nbscr.2025.100129 (PMC12151192; doi:10.1016/j.nbscr.2025.100129)
Supplement: Multimedia component 1 [file mmc1.docx]

**Supplementary Materials**

**Supplementary** **Figure 1: Covariate Balance Before and After Propensity Score Matching (Obstructive Sleep Apnea)**

**
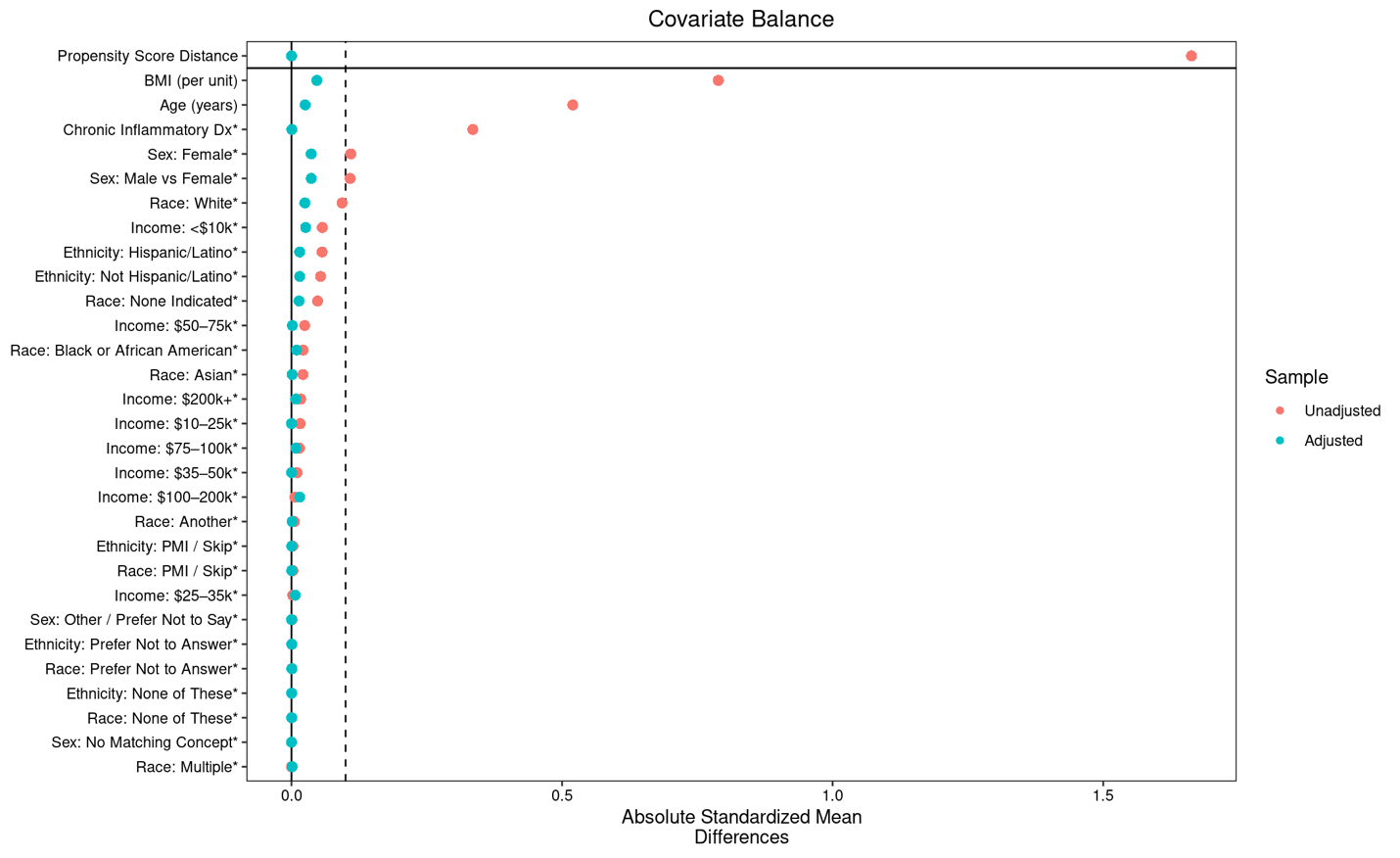
**

Standardized mean differences of covariates before and after 1:5 propensity score matching between participants with obstructive sleep apnea (OSA) and matched controls without OSA. Covariates include age, BMI, sex at birth, race, ethnicity, income level, and presence of chronic inflammatory diagnoses. The vertical dashed lines at ±0.1 denote the commonly accepted threshold for acceptable covariate balance. Matching substantially improved covariate balance across all variables.

**Supplementary** **Figure 2: Covariate Balance Before and After Propensity Score Matching (Hypersomnia)**

**
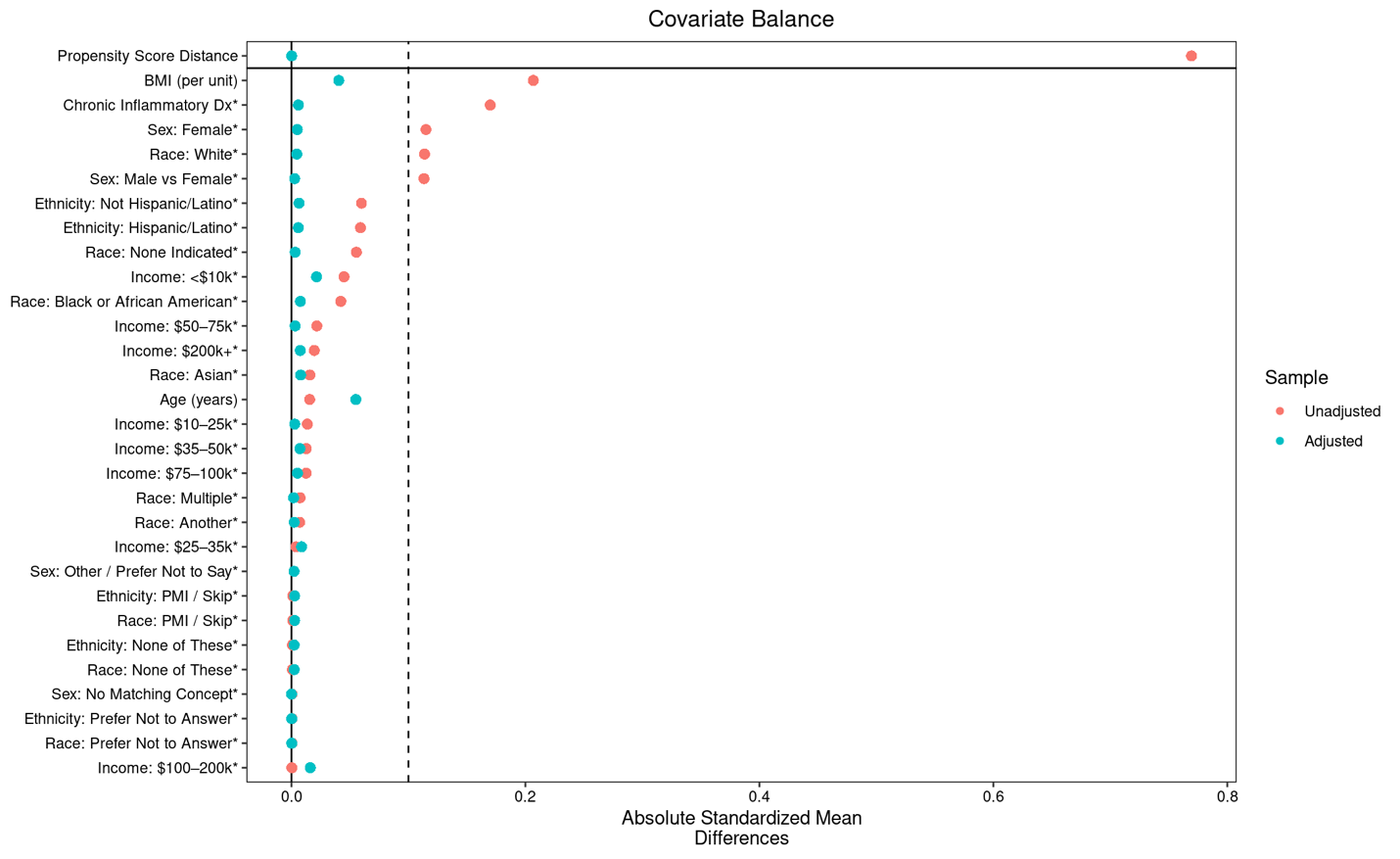
**

Standardized mean differences of demographic and clinical covariates before and after propensity score matching between participants with Hypersomnia (treated) and those without (control). Variables include age, sex at birth, race, ethnicity, household income, BMI, and chronic inflammatory diagnosis. The vertical dashed lines at ±0.1 represent the commonly accepted threshold for adequate covariate balance. Matching achieved improved balance across nearly all covariates.

### ****Supplementary Table 1. Adjusted Logistic Regression Results for Autoimmune Diagnosis (Rare Disease Design for Delayed Sleep Phase Disorder)****

| **Variable** | **Odds Ratio (OR)** | **95% CI Lower** | **95% CI Upper** | **FDR-Adjusted p-value** |
| --- | --- | --- | --- | --- |
| Chronic Inflammatory Dx: Yes * | 5.20 | 5.01 | 5.41 | < 2e-16 |
| Sex: Male vs Female * | 0.565 | 0.546 | 0.584 | < 2e-16 |
| Race: Black or African American * | 0.675 | 0.643 | 0.708 | < 2e-16 |
| Race: Asian * | 0.543 | 0.491 | 0.601 | < 2e-16 |
| Income: 100–200k * | 1.32 | 1.25 | 1.40 | < 2e-16 |
| Income: 200k+ * | 1.40 | 1.30 | 1.50 | < 2e-16 |
| Income: 10–25k * | 1.29 | 1.22 | 1.36 | < 2e-16 |
| Income: 50–75k * | 1.28 | 1.21 | 1.36 | < 2e-16 |
| Income: 75–100k * | 1.30 | 1.22 | 1.39 | < 2e-16 |
| Age (years) * | 1.00 | 1.00 | 1.00 | 1.0e-09 |
| Income: 35–50k * | 1.23 | 1.15 | 1.31 | 5.8e-06 |
| BMI (per unit) * | 0.994 | 0.991 | 0.996 | 1.9e-05 |
| Income: 25–35k * | 1.18 | 1.11 | 1.27 | 1.1e-04 |
| Chronotype: Regular vs Delayed * | 0.256 | 0.147 | 0.445 | 2.3e-04 |
| Race: None Indicated * | 0.803 | 0.728 | 0.885 | 0.0017 |
| Race: Another single population * | 0.809 | 0.719 | 0.910 | 0.0086 |
| Race: More than one population * | 0.880 | 0.818 | 0.946 | 0.0086 |
| Sex: Other / Prefer Not to Say * | 0.847 | 0.720 | 0.996 | 0.0385 |
| Race: I Prefer Not to Answer * | 0.774 | 0.598 | 1.00 | 0.0427 |
| Ethnicity: Not Hispanic or Latino | 1.08 | 0.985 | 1.17 | 0.104 |
| Race: None of These | 1.07 | 0.906 | 1.27 | 0.416 |
| Race: PMI Skip | 1.03 | 0.874 | 1.22 | 0.696 |
| Sex: No Matching Concept | 1.09 | 0.536 | 2.20 | 0.816 |

This table presents adjusted odds ratios (ORs), 95% confidence intervals (CIs), and Benjamini-Hochberg false discovery rate (FDR)–adjusted p-values from a logistic regression model evaluating the association between delayed sleep phase disorder (DSPD) and autoimmune diagnosis. The model includes covariates for age, sex at birth, race, ethnicity, income, chronic inflammatory diagnosis, and body mass index (BMI), based on a matched sample using a rare disease design. Odds ratios less than 1 indicate reduced odds of autoimmune diagnosis relative to the reference category. Reference groups: Sleep = Delayed, Sex = Female, Race = White, Ethnicity = Hispanic or Latino, and Income = <10k. Asterisks (*) denote statistical significance at FDR-adjusted p < 0.05.

### ****Supplementary Table 2. Adjusted Logistic Regression Results for Autoimmune Diagnosis Following Propensity Score Matching (Obstructive Sleep Apnea)****

| **Variable** | **Odds Ratio (OR)** | **95% CI Lower** | **95% CI Upper** | **Adjusted p-value** |
| --- | --- | --- | --- | --- |
| Chronic Inflammatory Dx: Yes * | 5.08 | 4.89 | 5.28 | < 2e-16 |
| Sex: Male vs Female * | 0.566 | 0.548 | 0.585 | < 2e-16 |
| Race: Black or African American * | 0.672 | 0.641 | 0.705 | < 2e-16 |
| Sleep: Regular vs OSA * | 0.461 | 0.411 | 0.517 | < 2e-16 |
| Race: Asian * | 0.549 | 0.497 | 0.606 | < 2e-16 |
| Income: 100–200k * | 1.31 | 1.24 | 1.39 | < 2e-16 |
| Income: 10–25k * | 1.30 | 1.23 | 1.37 | < 2e-16 |
| Income: 200k+ * | 1.37 | 1.28 | 1.46 | < 2e-16 |
| Income: 75–100k * | 1.33 | 1.24 | 1.41 | < 2e-16 |
| Income: 50–75k * | 1.28 | 1.21 | 1.36 | < 2e-16 |
| Age (years) * | 1.00 | 1.00 | 1.00 | 2.59e-15 |
| Income: 35–50k * | 1.22 | 1.15 | 1.30 | 7.54e-10 |
| BMI (per unit) * | 0.994 | 0.992 | 0.996 | 3.0e-08 |
| Income: 25–35k * | 1.19 | 1.12 | 1.27 | 1.18e-07 |
| Race: None Indicated * | 0.808 | 0.734 | 0.890 | 1.58e-05 |
| Race: More than one population * | 0.884 | 0.822 | 0.949 | 7.18e-04 |
| Race: Another single population * | 0.825 | 0.735 | 0.928 | 1.27e-03 |
| Sex: Other / Prefer Not to Say * | 0.839 | 0.715 | 0.984 | 3.14e-02 |
| Race: I prefer not to answer | 0.782 | 0.605 | 1.01 | 6.15e-02 |
| Ethnicity: Not Hispanic or Latino | 1.08 | 0.989 | 1.18 | 8.90e-02 |
| Race: None of These | 1.15 | 0.971 | 1.35 | 1.06e-01 |
| Sex: No Matching Concept | 1.22 | 0.615 | 2.40 | 5.74e-01 |
| Race: PMI: Skip | 1.01 | 0.855 | 1.19 | 9.10e-01 |

This table presents adjusted odds ratios (ORs), 95% confidence intervals (CIs), and Benjamini-Hochberg FDR–adjusted p-values from a logistic regression model evaluating the association between OSA diagnosis and autoimmune disease prevalence. The model includes covariates for age, sex at birth, race, ethnicity, BMI, income level, and chronic inflammatory diagnoses. It is based on a 1:5 matched rare disease design. Odds ratios less than 1 indicate reduced odds of autoimmune diagnosis compared to the reference category. Reference groups: Sleep = OSA, Sex = Female, Race = White, and Ethnicity = Hispanic. Asterisks (*) denote statistical significance at FDR-adjusted p < 0.05.

### ****Supplementary Table 3. Adjusted Logistic Regression Results for Autoimmune Diagnosis Following Propensity Score Matching (Rare Disease Design for Primary Insomnia)****

| **Variable** | **Odds Ratio (OR)** | **95% CI Lower** | **95% CI Upper** | **Adjusted p-value** |
| --- | --- | --- | --- | --- |
| Chronic Inflammatory Dx: Yes * | 5.08 | 4.89 | 5.28 | < 2e-16 |
| Sex: Male vs Female * | 0.566 | 0.548 | 0.585 | < 2e-16 |
| Race: Black or African American * | 0.672 | 0.641 | 0.705 | < 2e-16 |
| Sleep: Regular vs Primary Insomnia * | 0.461 | 0.411 | 0.517 | < 2e-16 |
| Race: Asian * | 0.549 | 0.497 | 0.606 | < 2e-16 |
| Income: 100–200k * | 1.31 | 1.24 | 1.39 | < 2e-16 |
| Income: 10–25k * | 1.30 | 1.23 | 1.37 | < 2e-16 |
| Income: 200k+ * | 1.37 | 1.28 | 1.46 | < 2e-16 |
| Income: 75–100k * | 1.33 | 1.24 | 1.41 | < 2e-16 |
| Income: 50–75k * | 1.28 | 1.21 | 1.36 | < 2e-16 |
| Age (years) * | 1.00 | 1.00 | 1.00 | 2.59e-15 |
| Income: 35–50k * | 1.22 | 1.15 | 1.30 | 7.54e-10 |
| BMI (per unit) * | 0.994 | 0.992 | 0.996 | 3.00e-08 |
| Income: 25–35k * | 1.19 | 1.12 | 1.27 | 1.18e-07 |
| Race: None Indicated * | 0.808 | 0.734 | 0.890 | 1.58e-05 |
| Race: More than one population * | 0.884 | 0.822 | 0.949 | 7.18e-04 |
| Race: Another single population * | 0.825 | 0.735 | 0.928 | 1.27e-03 |
| Sex: Other / Prefer Not to Say * | 0.839 | 0.715 | 0.984 | 3.14e-02 |
| Race: I prefer not to answer | 0.782 | 0.605 | 1.01 | 6.15e-02 |
| Ethnicity: Not Hispanic or Latino | 1.08 | 0.989 | 1.18 | 8.90e-02 |
| Race: None of These | 1.15 | 0.971 | 1.35 | 1.06e-01 |
| Sex: No Matching Concept | 1.22 | 0.615 | 2.40 | 5.74e-01 |
| Race: PMI: Skip | 1.01 | 0.855 | 1.19 | 9.10e-01 |

This table presents adjusted odds ratios (ORs), 95% confidence intervals (CIs), and Benjamini-Hochberg FDR–adjusted p-values from a logistic regression model evaluating the association between Primary Insomnia status and autoimmune diagnosis. The model includes covariates for age, sex at birth, race, ethnicity, BMI, income level, and chronic inflammatory conditions, using a rare disease design without matching. Odds ratios < 1 indicate lower odds of autoimmune diagnosis relative to the reference category. Reference groups: Sleep = Primary Insomnia, Sex = Female, Race = White, and Ethnicity = Hispanic. Asterisks (*) denote statistical significance at FDR-adjusted p < 0.05.

### ****Supplementary Table 4. Adjusted Logistic Regression Results for Autoimmune Diagnosis Following Propensity Score Matching (Hypersomnia)****

| **Variable** | **Odds Ratio (OR)** | **95% CI Lower** | **95% CI Upper** | **Adjusted p-value** |
| --- | --- | --- | --- | --- |
| Sleep: Regular vs Hypersomnia * | 0.482 | 0.464 | 0.500 | < 2e-16 |
| Sex: Male vs Female * | 0.560 | 0.545 | 0.576 | < 2e-16 |
| Chronic Inflammatory Dx: Yes * | 4.14 | 4.01 | 4.27 | < 2e-16 |
| Race: Black or African American * | 0.710 | 0.683 | 0.739 | < 2e-16 |
| Race: Asian * | 0.574 | 0.525 | 0.628 | < 2e-16 |
| Income: 10–25k * | 1.27 | 1.21 | 1.33 | < 2e-16 |
| Age (years) * | 1.00 | 1.00 | 1.00 | < 2e-16 |
| Income: 200k+ * | 1.29 | 1.22 | 1.37 | < 2e-16 |
| Income: 100–200k * | 1.23 | 1.17 | 1.29 | < 2e-16 |
| BMI (per unit) * | 0.993 | 0.991 | 0.995 | < 2e-16 |
| Income: 75–100k * | 1.23 | 1.16 | 1.29 | < 2e-16 |
| Income: 50–75k * | 1.20 | 1.14 | 1.26 | < 2e-16 |
| Income: 35–50k * | 1.15 | 1.09 | 1.22 | 3.20e-07 |
| Income: 25–35k * | 1.15 | 1.09 | 1.21 | 1.37e-06 |
| Race: None Indicated * | 0.828 | 0.761 | 0.902 | 2.20e-05 |
| Race: Another Single Population * | 0.815 | 0.737 | 0.901 | 9.30e-05 |
| Sex: Other / Prefer Not to Say * | 0.811 | 0.709 | 0.928 | 2.61e-03 |
| Race: More than one population * | 0.913 | 0.860 | 0.970 | 3.17e-03 |
| Race: I prefer not to answer | 0.861 | 0.697 | 1.06 | 0.163 |
| Ethnicity: Not Hispanic or Latino | 1.05 | 0.977 | 1.14 | 0.176 |
| Race: None of These | 1.08 | 0.934 | 1.24 | 0.310 |
| Sex: No Matching Concept | 0.882 | 0.498 | 1.56 | 0.667 |
| Race: PMI: Skip | 1.00 | 0.870 | 1.15 | 0.997 |

This table presents adjusted odds ratios (ORs), 95% confidence intervals (CIs), and Benjamini-Hochberg false discovery rate (FDR)–adjusted p-values from a logistic regression model evaluating the association between hypersomnia diagnosis and autoimmune disease prevalence. The model includes covariates for age, sex at birth, race, ethnicity, income, chronic inflammatory disease, and BMI, and is based on a 1:5 matched rare disease design. Odds ratios less than 1 indicate reduced odds of autoimmune diagnosis compared to the reference category. Reference groups: Sleep = Hypersomnia, Sex = Female, Race = White, Ethnicity = Hispanic or Latino, Income = <10k, Chronic Inflammatory Dx = No. Asterisks (*) denote statistical significance at FDR-adjusted p < 0.05.
